# Robust bioprocess design and evaluation of commercial media for the serial expansion of human induced pluripotent stem cell aggregate cultures in vertical-wheel bioreactors

**DOI:** 10.1186/s13287-024-03819-9

**Published:** 2024-07-29

**Authors:** Breanna S. Borys, Tiffany Dang, Hannah Worden, Leila Larijani, Jessica M. Corpuz, Brett D. Abraham, Emilie J. Gysel, Julia Malinovska, Roman Krawetz, Tamas Revay, Bob Argiropoulos, Derrick E. Rancourt, Michael S. Kallos, Sunghoon Jung

**Affiliations:** 1 Pharmaceutical Production Research Facility, University of Calgary, Calgary, AB Canada; 2 PBS Biotech Inc, 4721 Calle Carga, Camarillo, CA 93012 USA; 3 Department of Biomedical Engineering, University of Calgary, Calgary, AB Canada; 4 McCaig Institute for Bone and Joint Health, University of Calgary, Calgary, AB Canada; 5 Department of Medical Genetics, University of Calgary, Calgary, AB Canada; 6 Department of Biochemistry and Molecular Biology, University of Calgary, Calgary, AB Canada; 7 Department of Medical Genetics, Alberta Health Services, Alberta Children’s Hospital, Calgary, AB Canada; 8 Department of Cell Biology and Anatomy, University of Calgary, Calgary, AB Canada

**Keywords:** Stem cell bioprocessing, Induced pluripotent stem cell, Vertical-wheel bioreactor, Serial passage, Biomanufacturing, Media

## Abstract

**Background:**

While pluripotent stem cell (PSC) therapies move toward clinical and commercial applications at a rapid rate, manufacturing reproducibility and robustness are notable bottlenecks in regulatory approval. Therapeutic applications of PSCs require large cell quantities to be generated under highly robust, well-defined, and economically viable conditions. Small-scale and short-term process optimization, however, is often performed in a linear fashion that does not account for time needed to verify the bioprocess protocols and analysis methods used. Design of a reproducible and robust bioprocess should be dynamic and include a continuous effort to understand how the process will respond over time and to different stresses before transitioning into large-scale production where stresses will be amplified.

**Methods:**

This study utilizes a baseline protocol, developed for the short-term culture of PSC aggregates in Vertical-Wheel^®^ bioreactors, to evaluate key process attributes through long-term (serial passage) suspension culture. This was done to access overall process robustness when performed with various commercially available media and cell lines. Process output variables including growth kinetics, aggregate morphology, harvest efficiency, genomic stability, and functional pluripotency were assessed through short and long-term culture.

**Results:**

The robust nature of the expansion protocol was demonstrated over a six-day culture period where spherical aggregate formation and expansion were observed with high-fold expansions for all five commercial media tested. Profound differences in cell growth and quality were revealed only through long-term serial expansion and in-vessel dissociation operations. Some commercial media formulations tested demonstrated maintenance of cell growth rates, aggregate morphology, and high harvest recovery efficiencies through three bioreactor serial passages using multiple PSC lines. Exceptional bioprocess robustness was even demonstrated with sustained growth and quality maintenance over 10 serial bioreactor passages. However, some commercial media tested proved less equipped for serial passage cultures in bioreactors as cultures led to cell lysis during dissociation, reduction in growth rates, and a loss of aggregate morphology.

**Conclusions:**

This study demonstrates the importance of systematic selection and testing of bioprocess input variables, with multiple bioprocess output variables through serial passages to create a truly reproducible and robust protocol for clinical and commercial PSC production using scalable bioreactor systems.

## Introduction

One of the most urgent problems in regenerative medicine is a lack of suitable source of cells, tissues and organs used to replace or repair the biological function of damaged tissues. Pluripotent stem cells (PSCs) have generated significant attention owing to their capacity for self-renewal and ability to differentiate into all three germ layers. Once established, human PSC (hPSC) lines have almost unlimited proliferation capacity and can retain the ability to give rise to all cell lineages, making them an ideal platform material for cell-based therapies.

Successful implementation of a PSC-based therapy for clinical and commercial purposes will rely on the development of a robust and scalable cell culture process for the expansion and differentiation of these cells to enable production of a desired target number of specific cells (i.e., a manufacturing lot size to meet the clinical or commercial needs) in a consistent manner. PSCs are traditionally grown in static culture vessels as adherent monolayers or non-adherent spherical aggregates. Although sufficient to generate cells for experimental purposes, this approach is impractical to achieve large quantities required for clinical or commercial applications. For PSC-based treatments, cell dosages will range from 10^9^ to 10^12^ cells per patient depending on the therapeutic target [1]. To achieve the required number of cells in an effective manner, scalable bioreactors will need to be used. Biomanufacturing of cells for therapeutic purposes using such bioreactors is advantageous due to reduced operating and labor cost requirements, improved scalability, and the ability to fine-tune process control capabilities [2].

Suspension bioreactors have been employed by the bioprocessing industries for decades for mass production of recombinant proteins and monoclonal antibodies using well-established cell lines (such as Chinese Hamster Ovary cells) that grow well in dynamic liquid-mixing culture environments. However, suspension cultures introduce hydrodynamic forces which have been shown to impact various stem cell attributes such as proliferation and potency [3, 4]. Further, different suspension bioreactor geometries can modulate hydrodynamic environments differently which in turn impact these properties in unique ways depending on the geometry. Specifically, PSC cultures are shear sensitive and unfavourable environments adversely impact the growth and quality maintenance of PSCs [5]. Concerns surrounding DNA integrity are amplified when dealing with induced pluripotent stem cells (iPSCs) which are reported to have an increased risk over traditional embryonic stem cells of genetic and epigenetic abnormalities linked to the process of cell reprogramming [6, 7].

The Vertical-Wheel^®^ (VW) bioreactor family used in this study for the culture of human iPSCs (hiPSCs) has become increasingly popular as a platform for research and clinical studies of various modalities and the subject of computational fluid dynamic modelling and power measurement studies [5, 8–22]. This interest in the VW bioreactor stems from the unique geometry with the following features: (i) VW impeller and U-shaped vessel together promoting strong, sweeping liquid flow at the bottom of the bioreactor; (ii) oppositely oriented axial vanes creating cutting and folding fluid flow for enhanced mixing; and (iii) sizeable impeller zone having a large swept volume, which together creates a relatively uniform distribution of hydrodynamic forces making it an ideal tool for stem cell culture and bioprocess design [20].

Understanding bioprocess engineering fundamentals, including correlations between process input variables (PIVs), such as bioreactor design-related parameters, media components, feeding regime, harvest-related parameters, etc., and process out variables (POVs), including cell growth rate/yield, viability, aggregate size/morphology, phenotype, genomic stability, etc., need to become a focus in the development of next generation technologies and protocols capable of producing cell-based products in a safe and cost-effective manner.

**Fig. 1:**
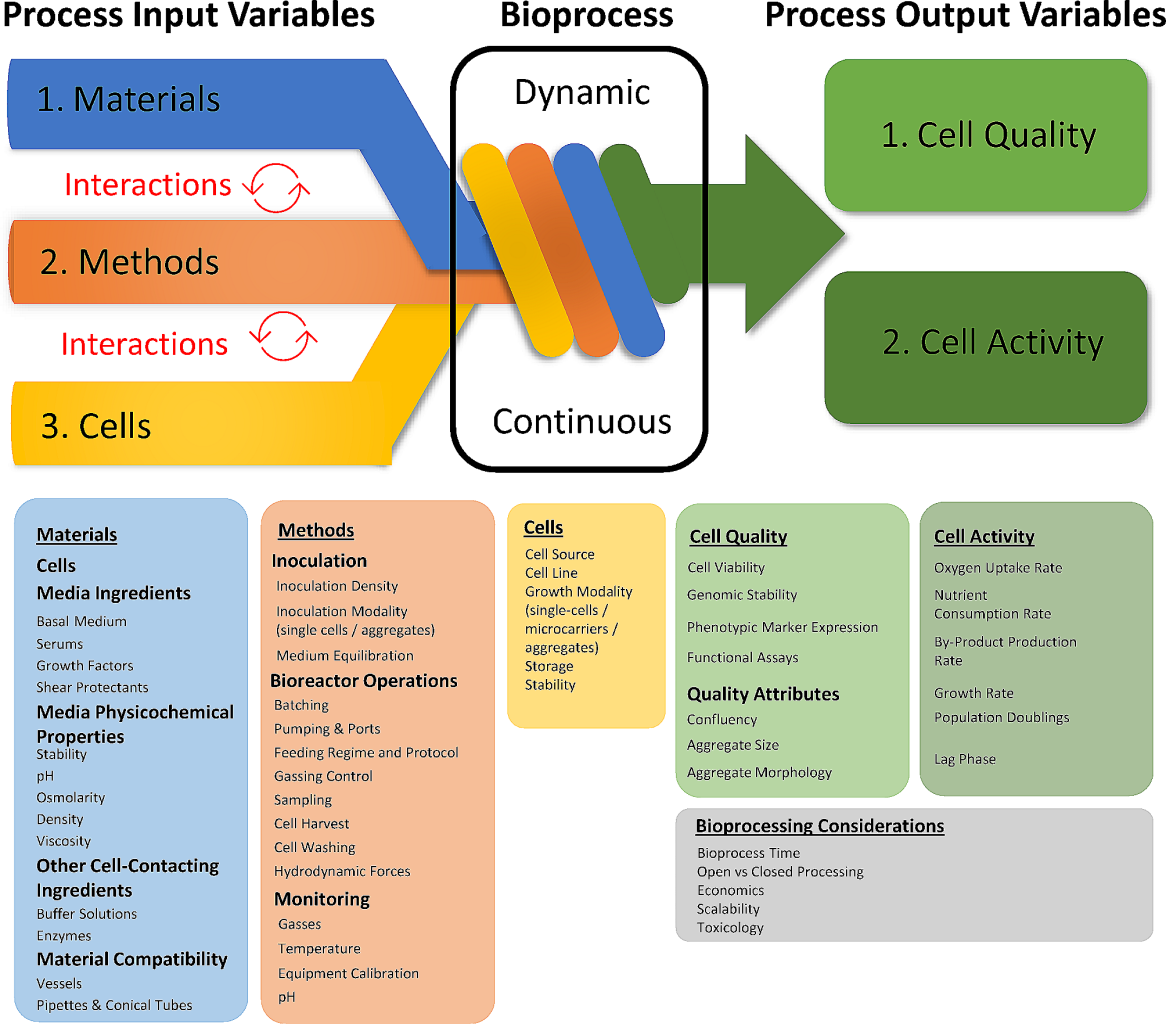
Bioprocess engineering graphic highlighting some examples of the process input variables (PIVs) and process output variables (POVs). When designing a bioprocess, it is important to consider critical PIVs and how they will impact a process independently or synergistically with other variables. Further, as a bioprocess is a continuous and dynamic process it is critical to understand how it will respond to different PIVs and the impact on key/critical POVs. Each box outlines various PIVs and POVs to consider when designing a process

While cell and gene therapies move towards clinical and commercial applications at a rapid rate, manufacturing concerns have been a notable source of products being held back from regulatory approval [23]. Therefore, key/critical POVs should include those related to manufacturing process predictability and consistency, also known as key process attributes, as well as critical quality attributes. Figure 1 highlights some of the impactful variables to be considered and analyzed within a stem cell bioprocess. The PIVs depicted in the diagram highlight just some of the variables to choose from when selecting materials, methods, and measurements to test in the design and analysis of a bioprocess. Each PIV can have a significant impact alone or interact with another variable on key/critical process and quality attributes. For example, material variables like buffer solutions, growth factors, and shear protectants are often tested at various concentrations individually or in concert to determine their impact on cell viability, cell growth rate, and phenotypic marker expression. Likewise, method variables like inoculation density, feeding regime, and agitation rate would also have a significant impact on output variables including cell growth rate, aggregate morphology, and aggregate size.

We have utilized the VW bioreactor in several studies [5, 20, 21] to culture hiPSCs as aggregates over multiple passages. The baseline bioprocess developed in these studies identified and systemically optimized key PIVs, such as agitation rate and environmental oxygen, and process protocols, such as inoculation and in-vessel aggregate dissociation, and achieved high cell fold expansion with minimal time and resource inputs. Creating a reproducible and robust bioprocess, however, should be a continuous and dynamic effort. It is important to test how the bioprocess will respond over time and to different stresses. The aim of the current study was to test the robustness of this baseline bioprocess for the short- and long-term expansion of hiPSCs in the VW bioreactor while assessing the impact of changing one of the most crucial PIVs – cell culture medium. Analysis of key POVs including cell growth rates, aggregate morphology, and harvest recovery provided insight into which commercially available media could be easily transitioned from a conventional planar method to a dynamic culture system for a serial passage of hiPSC culture. Importantly, when tested through one bioreactor passage all five media could have been considered acceptable, demonstrating similar cell expansion capabilities and suitable aggregate growth and morphology.

While this speaks to the robust nature of the bioprocess tested, it should also serve as a warning for conclusions drawn from short-term testing and analysis. For clinical and manufacturing cell and gene therapies, several bioreactor processes stages may be required. Process challenges were noted for three of the five commercial media tested during the serial passage with compounding impacts evident throughout the third bioreactor passage at which key process attributes were significantly reduced. The remaining two media tested using multiple hiPSC lines demonstrated consistent and robust serial expansion in the bioreactor with maintenance of cell growth rates, normal aggregate morphology, high harvest recovery efficiencies, genomic stability, and pluripotent function through three bioreactor serial passages. With use of one of these identified commercial media, this study lays the foundation for the transition of small-scale and short-term PSC protocols into robust bioprocesses with predictable key/critical process outputs over several bioreactor passages.

## Materials and methods

### Static culture of hiPSCs

In this study, three hiPSC cell lines were used: 4YA – derived from infant fibroblasts (University of Toronto, Toronto, Canada), TC1133 – derived from umbilical cord blood cells (Lonza, USA), and PLX1 – derived from the dermis of juvenile foreskin (Pluristyx, Seattle, USA). For all experiments, a static seed train consisting of two passages post-thaw were performed prior to bioreactor inoculation. Static culture was carried out on Matrigel-coated (Corning, 354277) T-75 flasks (ThermoFisher, 156800). To maintain consistency in cell quality going into the bioreactor cultures, all static seed trains were cultured using mTeSR1 medium (STEMCELL Technologies, 85850). From thaw, cells were seeded into T-75 flasks with mTeSR1 medium supplemented with 10 µM Y-27632 (STEMCELL Technologies, 72304) at a density of 15,000 cells/cm^2^ (0.32 mL/cm^2^ medium working volume). Full volume media exchanges were performed every 24 h with Y-27632-absent medium, and cells were passaged from the first static seed train stage to the second static seed train stage at 72 h. To passage the cells from static culture, the T-flasks were brought into the biosafety cabinet (BSC) where spent medium was removed from the T-flask using an aspirator. The static surface was washed two times with Ca^−^ and Mg^−^ PBS (Fisher Scientific, MT21031CV) (0.08 mL/cm^2^ per rinse). Accutase (STEMCELL Technologies, 07922) pre-heated to 37 °C and supplemented with 10 µM Y-27632 was added to the static flasks at an amount of 0.08 mL/cm^2^. The static cultures were placed back inside the incubator maintained at 37 °C in a humidified atmosphere with 5% CO_2_ for a minimum of 7 min. At 7 min, the culture flasks were brought out to be observed under the microscope to visually confirm cell detachment before proceeding. If a significant portion of cells appeared to still be attached (greater than ~ 10% of the population), the flask was placed back in the incubator for an additional 2 mins before it was visually checked again. Once cell detachment had been confirmed, the static flasks were brought back inside the BSC to collect the cells into centrifuge tubes. The cultures in the flasks were washed twice (0.08 mL/cm^2^ per wash) with mTeSR1 medium supplemented with 10 µM Y-27632, and the rinsed medium was added to the centrifuge tubes. The collected culture was centrifuged at 500 g for 5 min. Supernatant was then removed using an aspirator and the cell pellet was resuspended using a 5 mL pipette (Thomas Scientific, 1163Y21) containing 5.0 mL of fresh mTeSR1 medium supplemented with 10 µM Y-27632. The cell suspension was well-mixed and duplicate samples were removed to be counted using the NucleoCounter NC-200 (ChemoMetec) viable cell density protocol. Cells from the first static seed train stage were inoculated into the second static seed train stage at a density of 5,000 cells/cm^2^ (0.32 mL/cm^2^ medium working volume). Again, full volume media exchanges were performed every 24 h with Y-27632-absent medium. At this stage cells were passaged using the protocol described above at 88 h into bioreactor culture.

### Bioreactor culture of hiPSCs

In this study, the PBS-0.1 Mini single-use, VW bioreactor (PBS-0.1 Mini; PBS Biotech, FA-0.1-D-001) was used as a scale-down model of the VW bioreactor family. Prior to inoculation, PBS-0.1 Mini vessels were batched with 98% of the working volume of culture medium supplemented with 10 µM Y-27632 and placed on agitator bases, also called Mini bases, in the incubator (37 °C and 5% CO_2_) overnight. Five commercial PSC media were tested throughout this study for the expansion of hiPSCs in the bioreactors: mTeSR1 (STEMCELL Technologies, 85850), CTS E8 (ThermoFisher Scientific, A2656101), StemFlex (ThermoFisher Scientific, A3349401), PluriStem (Millipore Sigma, SCM130), and NutriStem (Sartorius, 05-100-1 A). Bioreactor culture protocols described in our previous publications [5, 21] were utilized. Briefly, hiPSCs harvested from the second stage of the static seed train were inoculated as a single cell suspension into the PBS-0.1 Mini at a density of 20,000 cells/mL. Throughout the culture, the impeller agitation rate was maintained at 40 rpm. A 50% medium exchange (MX) was performed on day 4 of culture with Y-27632-absent medium. For the MX procedure, agitation was stopped inside the incubator for 5 min to allow aggregates to settle. The bioreactor was then carefully brought into the BSC where 50% of the working volume of medium was removed from the top of the liquid volume and dispensed into a waste container. The equivalent volume of fresh, pre-warned medium was immediately added back into the bioreactor. The bioreactor was then transferred back into the incubator onto the Mini base.

### Bioreactor cell counts and aggregate sizing

Throughout the culture periods, samples taken from the bioreactor were used for cell counts to assess growth kinetics and phase-contrast imaging to assess aggregate morphology and size. Prior to sampling, Accutase aliquots were warmed in a 37 °C water bath for 20–40 min. To sample, the PBS-0.1 Mini was brought into the BSC and placed onto a Mini base operating between 40 and 60 rpm. The agitation rate for sampling was high enough that sufficient mixing of aggregates was visually noticeable. This was especially important to help ensure a representative sample was taken on later days of culture when larger aggregates required an increase in agitation to be fully mixed during sampling. Once the PBS-0.1 Mini was placed onto the base, the vessel cap was carefully removed and a 5 mL serological pipette was used to aspirate a 3.0 mL sample for cell counts, dispensed into a 15 mL conical tube (Thomas Scientific, 1207N11), followed by a 1.0 mL sample for aggregate imaging, dispensed into a single well of a 6-well plate (Thomas Scientific, 6902D01). The 15 mL conical tube was centrifuged at 500 g for 5 min. Supernatant was removed using a P1000 micropipette and 1.0 mL of pre-warmed Accutase was added to the cell pellet. The addition resuspended the aggregates without trituration. The cell tube was then placed into the 37 °C water bath for 20 min. During this time, the tube was removed every 5 min, flicked to resuspend the aggregates in the Accutase, and placed back in the water bath. This procedure was employed to avoid trituration that could cause cell loss or cell damage during the dissociation period while the flicking motion would facilitate a gentle dissociation as the aggregates resettle quickly into a pellet. At the 20 min mark (i.e., when the cell aggregates had been partially dissociated into loose clumps), the sample was triturated with a P1000 micropipette and transferred to a 1.7 mL microcentrifuge tube (Corning, MCT-175-C-S) for counting. The sample was counted using the NC-200 viability and cell count assay. Before each sample was counted, the microcentrifuge tube was vortexed (Fisher Scientific, 02215365) for 3 s at setting 8. Three counts were taken per bioreactor sample and averaged. Operators were not blinded to the different conditions while performing cell counts. In this study, the cumulative growth rate was calculated using Eq. 1:

1 $$\mu =\frac{LN \left(\raisebox{1ex}{${X}_{t}$}\!\left/ \!\raisebox{-1ex}{${X}_{0}$}\right.\right)}{t}$$

Where µ was the ‘overall’ apparent specific growth rate (hr^− 1^), X_0_ was the viable cell/mL target seeding density at inoculation time, in this case 20,000 cells/mL, and X_t_ was the average viable cell/mL density from sample counts at the end of culture time (t). The cumulative multiplication over the 3 bioreactor serial passages was calculated using Eq. 2:

2 $$\eqalign{ Cumulative{\mkern 1mu} Multiplication \cr & = \left( {{X_{P1}}{\mkern 1mu}/{X_0}} \right)\; \times \left( {{X_{P2}}{\mkern 1mu}/{X_0}} \right)\; \times \;\left( {{X_{P3}}{\mkern 1mu}/{X_0}} \right) \cr}$$

Where variables X_P1_, X_P2_, and X_P3_ were average viable cell/mL densities from sample counts at the end of bioreactor passage 1 (P1), end of bioreactor passage 2 (P2), and end of bioreactor passage 3 (P3), respectively.

Aggregates samples were imaged using a Nikon Eclipse Ts2-FI microscope, and the NIS-Elements software was used for measurements. Aggregates were defined as multi-cellular spheroids with a diameter greater than 50 μm. The diameter for each aggregate was calculated by taking the average of the greatest length across the aggregate and the length perpendicular to the greatest length. Again operators were not blinded to the different conditions while aggregate sizing.

### Cell harvest via in-vessel Dissociation of aggregates in bioreactors and serial passage

In-vessel aggregate dissociation and bioreactor-to-bioreactor serial passaging protocols were adapted from our past publication [5]. Briefly, the agitation was stopped inside the incubator for 5 min to allow aggregates to settle. The bioreactor was then carefully brought into the BSC where a 50 mL serological pipette was used to remove the first portion of the spent medium. Once the medium volume fell below the bioreactor wheel, a 10 mL serological pipette was used to remove the remaining liquid volume while carefully leaving the settled aggregate culture and approximately 1.0 mL of medium inside the bioreactor. Next, 20.0 mL of pre-warmed Accutase supplemented with 10 µM Y-27632 was added to the bioreactor. The bioreactor was brought back inside the incubator and placed on the Mini base at 80 rpm for a period of 20 min. After 20 min, the bioreactor was brought back inside the BSC where a 10 mL serological pipette was used to transfer the bioreactor contents into a 50 mL conical tube. Then, 20.0 mL of fresh medium supplemented with 10 µM Y-27632 was added to the conical tube to dilute the enzyme. The collected cells were centrifuged at 500 g for 5 min. Post centrifugation, the diluted enzyme was removed using an aspirator, and the cell pellet was resuspended using a 5 mL pipette containing 5.0 mL of fresh medium supplemented with 10 µM Y-27632. Another 5.0 mL of supplemented medium (for a total of 10.0 mL) was added to the resuspended cells to dilute the cell suspension appropriately for counts. The dissociated cell suspension was then well-mixed, and a 5 mL serological pipette was used to remove a 0.5 mL sample into a 6-well plate for microscopic imaging and 2 × 0.5 mL samples into microcentrifuge tubes for cell counts using the NuceloCounter NC-200 viable cell density protocol. These counts were used to calculate the inoculation volume required to seed the serial bioreactor culture at 20,000 cells/mL. Operators were not blinded to the different conditions while performing the in-vessel dissociations.

In this study, the harvesting efficiency was used to estimate the number of cells recovered from the harvesting process. The harvesting efficiency was defined using Eq. 3:

3 $$Harvest\,Efficiency = \,{B \over A}\, \times \,100\%$$

In the above equation variable B is defined as the total number of cells recovered from the cell harvest process (post harvest) and variable A is the total number of cells before the in-vessel dissociation procedure (pre harvest). The value of variable A (the total number of cells pre harvest) was obtained from the daily sample cell counts prior to cell harvest. Here the aggregates were dissociated in a traditional manner for daily cell counts as described earlier. The culture working volume prior to harvest was the sum of the medium volume removed prior to addition of Accutase used for the in-vessel dissociation plus the remaining amount of medium with aggregates settled on the bottom of the bioreactor (approximately 1.0 mL). Variable A can be summarized as Eq. 4:

4 $$\eqalign{& A = Ave{\mkern 1mu} \,{{cell} \over {mL}}\,{\mkern 1mu} bioreactor\,{\mkern 1mu} sample\,{\mkern 1mu} count \cr & \quad \, \times \,reactor{\mkern 1mu} \,volume{\mkern 1mu} \,pre{\mkern 1mu} \,harvest \cr}$$

The value of variable B (the total number of cells post harvest) was obtained using the aforementioned method in this section. Specifically, 2 × 0.5 mL samples from the resuspended cell suspension was aliquoted into microcentrifuge tubes for cell counts. The volume post dissociation is the volume of media used to resuspend the final cell suspension. Variable B can be summarized as Eq. 5:

5 $$\eqalign{ B \cr & = Ave{\mkern 1mu} \,{{cell} \over {mL}}\,{\mkern 1mu} cell\,resuspension\,sample\,count\,{\mkern 1mu} \cr & \times {\mkern 1mu} \,resuspension\,volume\,{\mkern 1mu} post{\mkern 1mu} \,harvest \cr}$$

### Karyotyping

For quality testing, cryopreserved cell samples from the bioreactor serial passage (StemFlex medium, bioreactor day 60) were thawed into T-75 flasks for recovery to be used for karyotyping and teratoma testing. For karyotyping, when the cells reached 60–70% confluency the medium was supplemented with 0.1 mg/mL KaryoMax Colcemid (Thermo Scientific, 15212012) for 4 h. The cells were then enzymatically dissociated as previously described. Single cells were collected by centrifugation, suspended in 0.075 M KCl hypotonic solution (Fisher Scientific, P217-500) and incubated at 37 °C for 25 min. Cells were then fixed with 3:1 methanol: acetic acid solution (Fisher Scientific, A412-4; EMD, AX0073) and chromosome preparations were GTG-banded using standard cytogenetic techniques. Karyograms were analyzed according to the ISCN standards at ~ 450 band resolution using the Ikaros karyotyping system (Metasystems).

### Teratoma formation

Teratoma formation assay was performed in accordance with animal protocol AC19-0134 approved by the University of Calgary animal care committee. One million dissociated hiPSCs in in 100 µL of PBS containing 10 µM Y-27632 were injected subcutaneously into the right and left dorsal flanks of 8–10-week-old female SCID Beige mice. All animals were housed under a standard light cycle and had access to food and water *ad libitum*. Prior to injection of human pluripotent cells, mice were anaesthetized under isoflurane (Baxter) anesthesia (1.5% v/v O_2_) for the duration of the procedure. At the time of sacrifice, mice were anaesthetized under isoflurane followed by cervical dislocation as approved under the animal protocol. After six weeks of tumor growth following implantation, mice were sacrificed. The development of the teratoma was monitored visually throughout the length of the experiment and if any tumor was over 1.0 cm in diameter (determined by palpation) this was considered to be a humane endpoint. Following dissection, tumors were fixed in 5% paraformaldehyde for 24 h, processed in an automated tissue processing system, paraffin-embedded and sectioned. Sectioned were placed on microscope slides to be stained with hematoxylin/eosin, imaged using the Zeiss Axio Scan microscope (Zeiss, Germany) and evaluated for the presence of tissues representative of the three embryonic germ layers. Only the individual dissociating the hiPSCs was aware of the different conditions. Individuals conducting the teratoma formation assay, outcome assessment, and data analysis of the germ layers were blinded to the specific conditions.

### Immunohistochemistry

10 μm paraffin-embedded sections were treated with CitriSolv (Fisher Scientific, 04-355-121) and rehydrated using a series of ethanol solutions. Antigen retrieval was conducted using a 10 mM sodium citrate solution at pH 6.0. Blocking was conducted using 5% BSA in PBS for 1 h. Primary antibodies were incubated overnight with: anti-beta III Tubulin (Abcam, ab18207), anti-GATA4 (Abcam, ab84593), recombinant anti-brachyury (Abcam, ab209665), and anti-human nuclear antigen antibody [235-1] (Abcam, ab191181). This was followed by a second overnight staining step with: goat anti-mouse IgG with Alexa Fluor™ 568 (Thermo Scientific, A-11031), donkey anti-rabbit IgG with Alexa Fluor™ 647 (BioLegend, 406414), and Hoechst 33342 (Thermo Scientific, H3570) for nuclear counterstaining and coverslipped.

### Reverse transcription quantitative polymerase chain reaction (RT-qPCR)

Pluripotency was assessed through real time quantitative polymerase chain reaction (RT-qPCR). RNA was collected from each sample using TRIzol Reagent (Invitrogen, 15596026) and converted to cDNA using a High Capacity cDNA kit (Applied Biosystems, 4368814,). The cDNA was then probed using Taqman validated primers for human Oct4 and Sox2 on a QuantStudio 6 Flex Real-Time PCR System (Applied Biosystems) using the housekeeping gene GAPDH as an internal control. The Taqman primers are summarized in Table 1. The ddCT method was then used to analyze the results.

**Table 1:** Primers used for RT-qPCR

Marker	Source, catalog #	Assay ID	Species
OCT4	ThermoFisher, 4331182	Hs00999632_g1	Human
SOX2	ThermoFisher, 4331182	Hs04234836_s1	Human
GAPDH	ThermoFisher, 4331182	Hs02758991_g1	Human

### Statistics

Statistical analysis was completed using GraphPad Prism Version 9 (Dotmatics). A one-way ANOVA followed by a Tukey’s multiple comparison test was used for significance testing for both fold expansion and aggregate size comparisons. The minimum P value was set to be 0.05. For the initial studies focused on short and long-term expansion, samples were collected in duplicate for *n* = 4 stirred suspension bioreactors. For the serial expansion studies using the TC1133 and PLX1 cell lines, samples were collected in duplicate for *n* = 2 stirred suspension bioreactors. For RT-qPCR, a one-way ANOVA followed by a Tukey’s comparison test was performed. Again, the minimum P value was set to be 0.05. The error bars on all graphs represent the ± standard error of the mean (SEM).

## Results

### Media screening in bioreactor culture

**Fig. 2:**
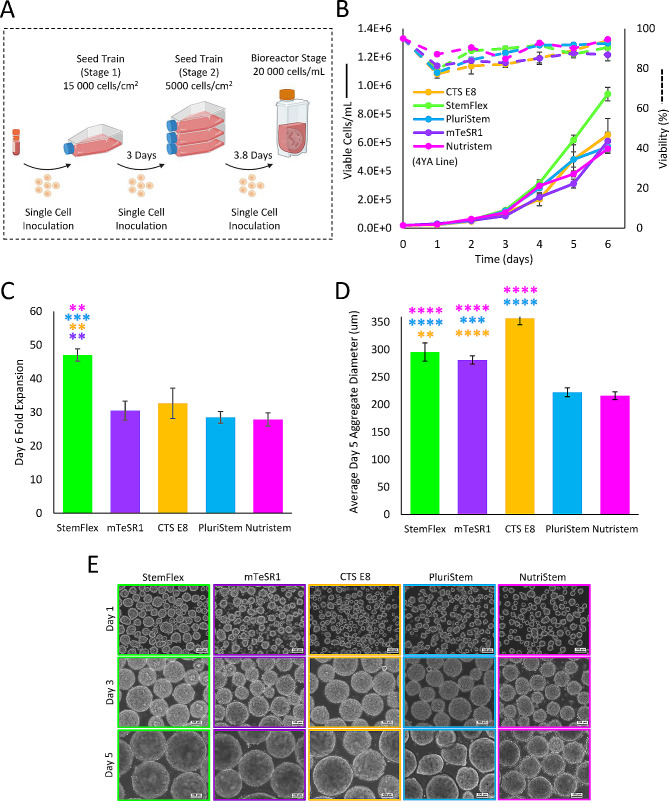
Static seed train used to initially expand hiPSCs before inoculation into 0.1 L Mini Vertical-Wheel bioreactors (**A**). Six-day growth curves for hiPSCs cultured in 0.1 L Minis (**B**) with Day 6 fold expansion (**C**) for each medium. (P values for StemFlex to NutriStem = 0.0072, StemFlex to PluriStem = 0.0004, StemFlex to CTS E8 = 0.0080, StemFlex to mTeSR1 = 0.0017. All other comparisons were not significant.). Average Day 5 aggregate diameters (**D**) with representative phase contrast microscope images (10X magnification) (**E**) for each medium on Days 1, 3, and 5 of culture. (P values for StemFlex to NutriStem < 0.0001, StemFlex to PluriStem < 0.0001, StemFlex to CTS E8 = 0.0029, mTeSR1 to NutriStem < 0.0001, mTeSR1 to PluriStem = 0.0001, mTeSR1 to CTS E8 < 0.0001, CTS E8 to NutriStem < 0.0001, CTS E8 to PluriStem < 0.0001. All other comparisons were not significant.) Statistics legend: **, ***, **** represent *P* ≤ 0.01, *P* ≤ 0.001, and *P* ≤ 0.0001, respectively

To initially assess bioprocess robustness, five commercial media were tested in the PBS-0.1 Mini bioreactors, including CTS E8, StemFlex, PluriStem, mTeSR1, and NutriStem. The cells were expanded according to the seed train protocol outlined in Fig. 2A, using mTeSR1 medium in T-flasks. These cells were then subsequently inoculated into the PBS-0.1 L Mini bioreactors as single cells and expanded for a six-day period. The process input variables for all bioreactors were the same except for the media type used and were adapted from our previous publication [5]. Under these conditions, all tested media were able to successfully cultivate hiPSC aggregates in the bioreactors. This was assessed by two criteria: (i) spherical aggregate morphology with smoothed and defined edges and lack of translucent cyst like structures; and (ii) cell growth with no prolonged lag phase and growth rates comparable to maxima found in current literature. The latter was supported for all tested media (Fig. 2B). StemFlex resulted in the greatest fold-expansion of 47.0 ± (1.8) in 6-days whereas other media resulted in ~ 30 fold-expansion in 6-days, as shown in Fig. 2C. Regarding aggregate morphology, the average diameter of aggregates varied depending on the media tested; CTS E8 medium demonstrated the greatest average aggregate diameter while PluriStem and NutriStem resulted in the smallest **(**Fig. 2D**)**. Less spherical morphology was also observed at a late time point in PluriStem medium while other media resulted in spherical morphology throughout the culture period **(**Fig. 2E**)**.

### In-vessel dissociation of aggregates following expansion in bioreactor

**Fig. 3:**
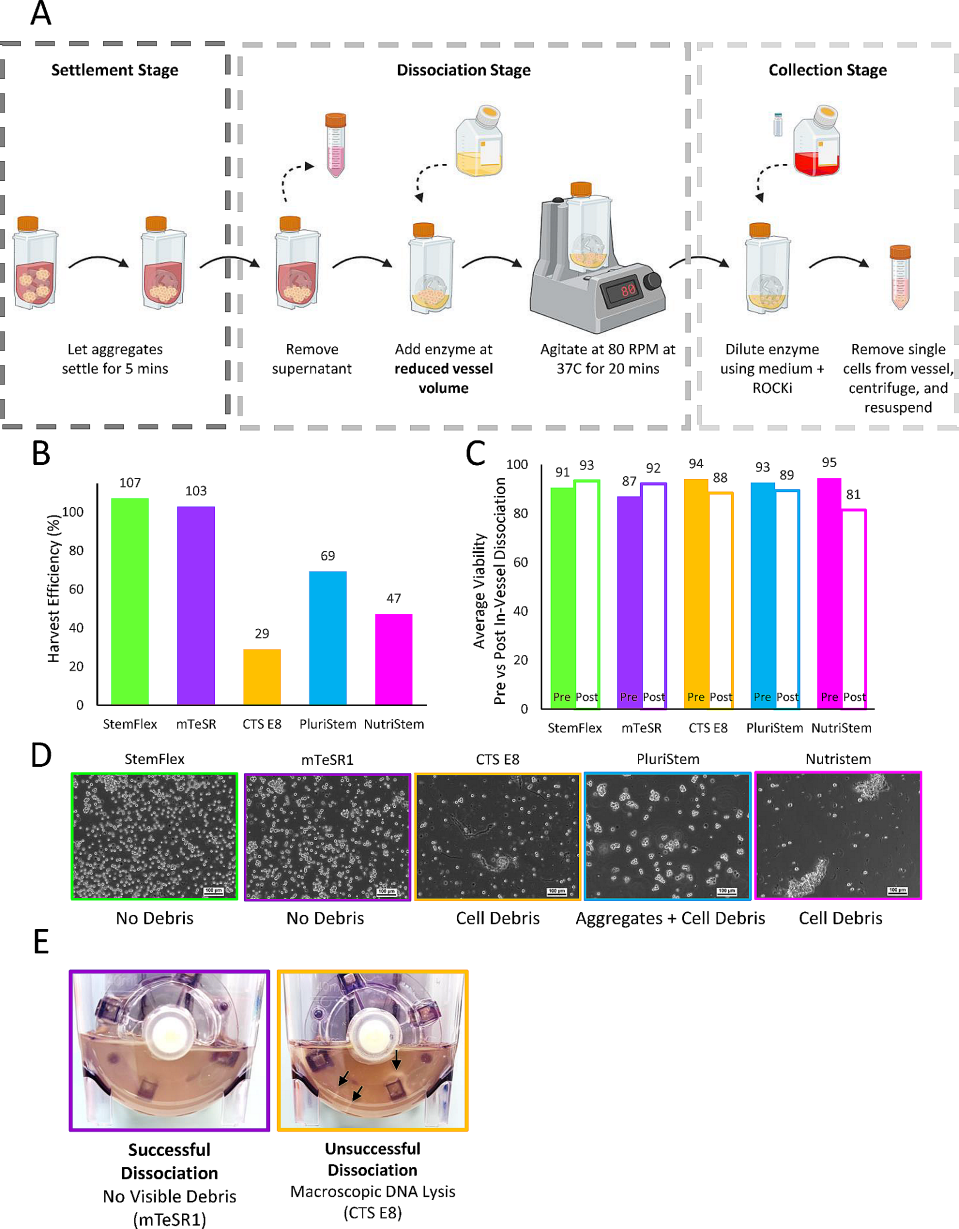
Schematic of the in-vessel dissociation protocol used to dissociate the aggregates into single cells on Day 6 of culture (**A**). Harvesting efficiencies following the in-vessel dissociation process (**B**) with the average viability pre and post dissociation for each medium (**C**). Representative phase contrast microscope images (10X magnification) of the cell suspension following the dissociation protocol for each medium (**D**). Comparison of successful dissociation (mTeSR1 on the left panel) and unsuccessful dissociation (CTS E8 on the right panel) (**E**)

Following the six days of expansion, an in-vessel aggregate dissociation protocol was employed using a method adapted from our previous work [5] for all tested media. A schematic of this protocol is shown in Fig. 3A. Although all tested commercial media were successfully expanded in the PBS-0.1 Mini bioreactors, profound differences in cell quality were observed following the use of the in-vessel aggregate dissociation protocol. As seen in Fig. 3B, high harvesting efficiencies were achieved from the cell aggregates that had been cultured in StemFlex and mTeSR1, demonstrating ~ 100% recovery. Conversely, low harvesting efficiencies were achieved from the cultures with CTS E8, PluriStem, and NutriStem at 29%, 69% and 47% respectively. For the cases where high in-vessel dissociations were observed (mTeSR1 and StemFlex), the post-dissociation cell viability remained above 90%, which was comparable to the sample count cell viability (Fig. 3C**)**. It should be noted that while the cell counts from CTS E8, PluriStem and NutriStem conditions were significantly lower post in-vessel dissociation, the cell viabilities did not display the same significant drop, measuring 88%, 89% and 81% by the automated NC-200 cell analyzer, respectively. This may indicate that viability alone (i.e., at least the viability measured by the method used in the present study) is not a good measure of cell health for downstream processing.

In addition to differences in harvesting efficiency, the impact on cell quality following the dissociation protocol was visualized using a brightfield microscope. From the images shown in Fig. 3D, the aggregates that had been grown in StemFlex and mTeSR1 in the PBS-0.1 Mini bioreactors were successfully dissociated into mostly single cells with minimal cell debris visible microscopically or macroscopically. However, use of the same aggregate dissociation protocol resulted in many poorly dissociated aggregates and cellular debris in CTS E8, PluriStem, and NutriStem conditions. This was further evident when macroscopically comparing the supernatant within the bioreactor after the 20 min of agitation time. Figure 3E displays representative images of what was observed during successful in-vessel dissociations and unsuccessful dissociations. Here, the mTeSR1 condition was shown as a successful example of in-vessel dissociation, in which the Accutase solution had become more turbid because of the concentrated single-cells from the aggregates dissociating. There were no macroscopically visible cell aggregates remaining or cell debris formed. In the case of the CTS E8 condition, the Accutase was less turbid with single-cells, and there were numerous visible strings of cell debris attached to sticky DNA indicative of cell lysis.

### Bioreactor serial passages

**Fig. 4:**
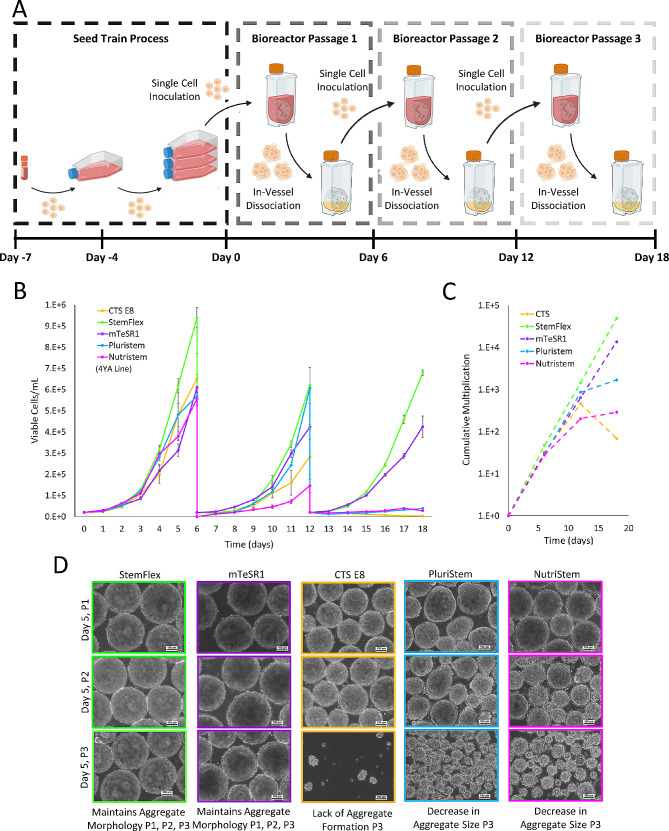
Schematic of the process used to passage cells for three serial passages from the seed train stage to the end of third passage in the 0.1 L Minis (**A**). Growth curves for three serial passages (**B**) and cumulative multiplication ratio (**C**) for each tested medium. Representative phase contrast microscope images (10X magnification) for each medium on Day 5 of Passage 1 (P1), Passage 2 (P2), and Passage 3 (P3) (**D**)

After initial screening in one bioreactor passage (short term culture), the five different commercially available media were also assessed after three serial passages (long term culture). A schematic of this process is outlined in Fig. 4A. Briefly, cells were expanded in static culture before inoculating PBS-0.1 Mini bioreactors using the same single-cell inoculation strategy as previously described. Following six days of bioreactor culture, the aggregates were dissociated using the in-vessel dissociation protocol. The cells from this dissociation were then used to inoculate the next set of bioreactors using the same single-cell inoculation method. To assess bioprocess impacts over several passages, cell growth and aggregate morphology were analyzed. As shown in Fig. 4B, StemFlex and mTeSR1 media resulted in the most consistent cell growth at each passage. Conversely, CTS E8, PluriStem, and NutriStem resulted in decreased cell growth in subsequent passages and negligible growth by the third bioreactor passage. The use of both StemFlex and mTeSR1 media maintained high cell fold-expansion during the serial passage (Fig. 4B), resulting in a cumulative multiplication of 4.95E4 and 1.37E4 over 18 days, respectively (Fig. 4C). In addition to cell growth and recovery, aggregate morphology was assessed during the three serial passages as shown in Fig. 4D. Like the growth kinetics results, the use of StemFlex and mTeSR1 media resulted in normal spherical aggregate morphology at the end of each of the three bioreactor passages. Conversely, changes in aggregate size and morphology were observed for CTS E8, PluriStem, and NutriStem conditions starting in the second passage. Of note, the microscope images are only representative of aggregate size and not of aggregate concentration (i.e., number of aggregates per unit volume) since the microscope field of view was too small to capture all the aggregates in each sample. Although there was macroscopically evident cell lysis and correspondingly low harvest recovery rates noted during the in-vessel dissociation of aggregates grown in CTS E8, PluriStem, and NutriStem media, the cells did show some recovery in terms of growth during the second bioreactor passage. The morphological changes and decrease in aggregate size along with the loss of proliferative capacity were most evident in the third bioreactor passage.

### Extended bioreactor serial passage and quality testing

**Fig. 5:**
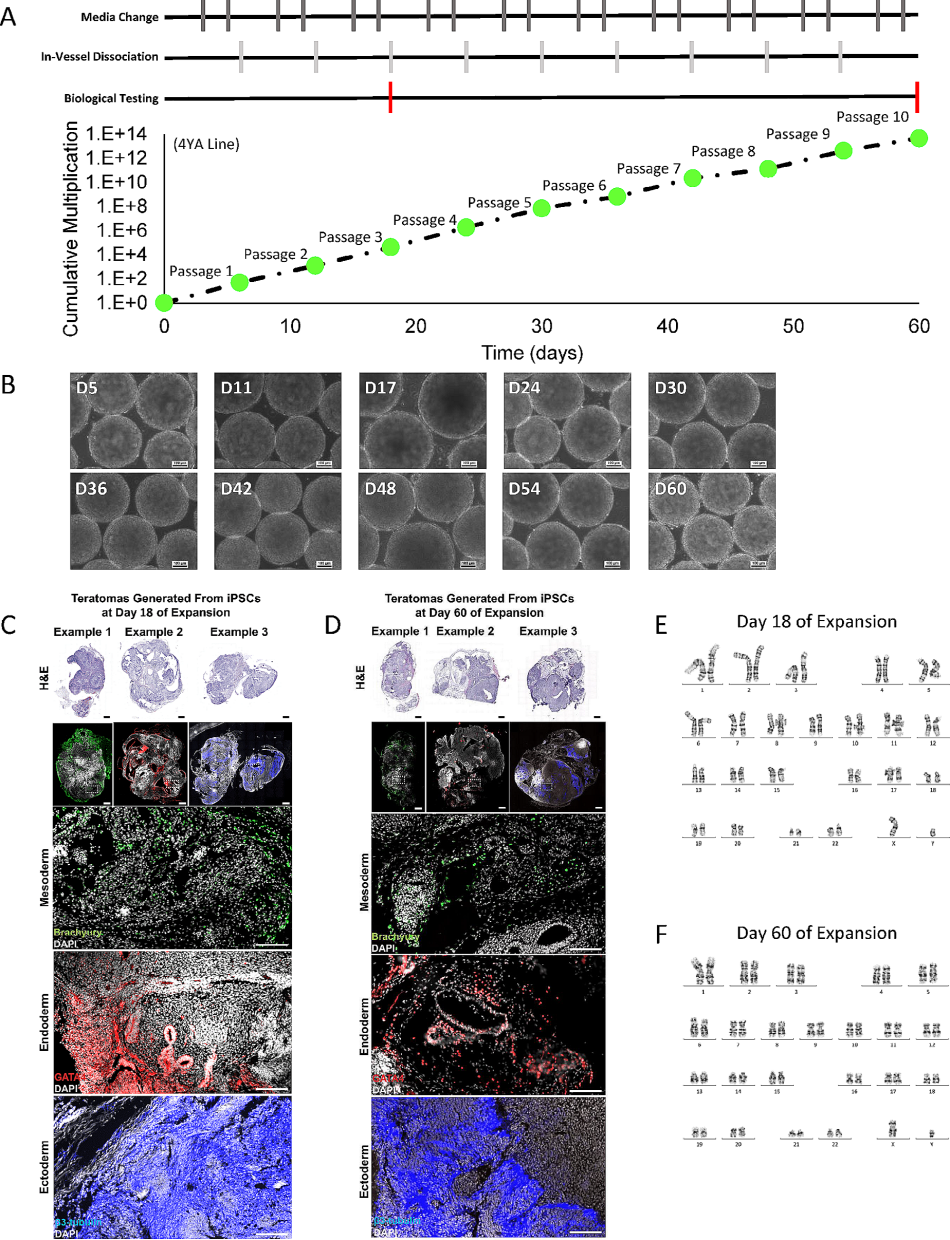
Cumulative multiplication ratio for hiPSCs cultured over ten serial passages in StemFlex medium with timeline of when media changes, in-vessel dissociations, and biological testing (both karyotyping and teratoma formation) were performed (**A**). Representative phase contrast microscope images (10X magnification) at the end of each passage (**B**). Biological testing via teratoma formation was performed at the end of the third (**C**) and tenth passage (**D**) with formation of tissue from all three germ layers indicating pluripotent function in vivo. This was further confirmed using immunohistochemistry of the resulting teratomas confirming the formation of the three germ layers (**C** and **D**, respectively). G-banding karyotyping was also performed at the end of the third (**E**) and tenth passage (**F**)

From the short- and long-term media screening, StemFlex was selected to assess protocol robustness and hiPSC biological quality through ten serial passages (60 days). The same process using single-cell inoculation and in-vessel aggregate dissociation was used to perform this screening in the PBS-0.1 Mini bioreactors. Figure 5A outlines when media replenishment, passaging, and biological testing were performed during this experiment. As shown in Fig. 5A, cell growth rate was well maintained during the ten serial passages in bioreactors, achieving a cumulative fold expansion of 4.62E13 in 60 days. Further, consistent and spherical aggregate morphology was observed at the end of each passage as shown in Fig. 5B indicating the long-term reproducibility and robustness of the developed process using the VW bioreactors. In addition to the analysis of growth kinetics and aggregate morphology, samples were taken at the end of passage three (day 18) and passage ten (day 60) to assess maintenance of functional pluripotency and genomic stability. After three serial passages in the bioreactors, the in vivo teratoma analysis confirmed functional pluripotency. This was further confirmed via immunohistochemistry (IHC) staining for markers representative of the three germ layers show in Fig. 5C. This teratoma formation assay along with IHC were also performed at the end of the tenth passage. This assay also confirmed the presence of a functional pluripotent population of cells which gave rise to all three germ layers shown in Fig. 5D. Further, karyotyping analysis demonstrated genetic stability at the end of the third and tenth passage as shown in Fig. 5E and F, respectively.

### Validating protocol robustness using additional hiPSC lines

**Fig. 6:**
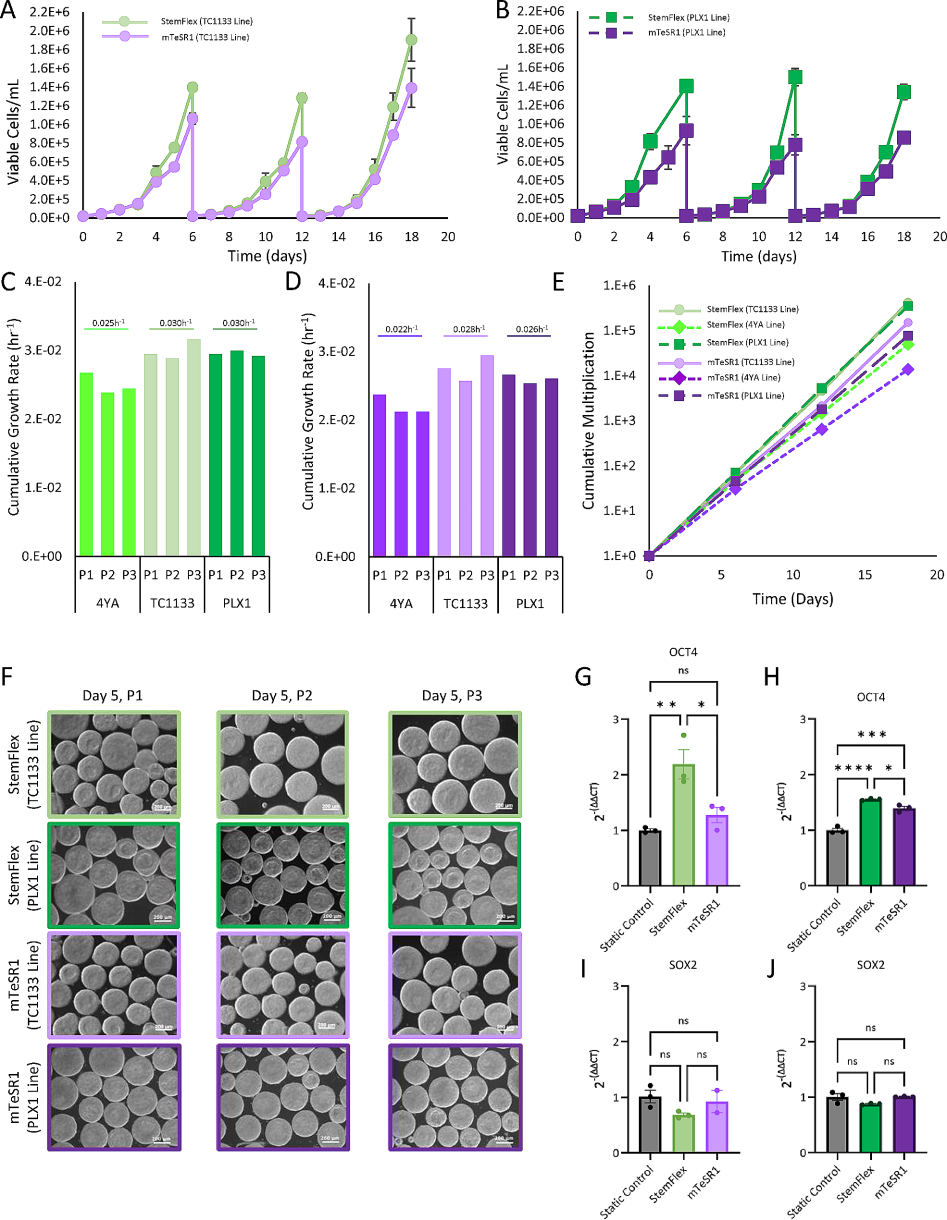
Growth curves for hiPSC line TC1133 (**A**) and PLX1 (**B**) cultured for three serial passages in both StemFlex and mTeSR1 media. Cumulative growth rate at each passage for all tested hiPSC lines in this study in StemFlex (**C**) and mTeSR1 (**D**). Cumulative multiplication ratio for all tested hiPSC lines in either StemFlex and mTeSR1 (**E**). Representative phase contrast microscope images (10X magnification) for TC1133 and PLX1 aggregates cultured in either StemFlex or mTeSR1 on Day 5 of Passage 1 (P1), Passage 2 (P2), and Passage 3 (P3) (**F**). Quantitative PCR evaluation of pluripotency associated genes Oct4 for the TC1133 line (**G**) and PLX1 (**H**) and Sox2 for the respective lines (**I** and **J**). TC1133 and PLX1 hiPSCs cultured in static conditions (Static Control) were used as controls to compare relative expression to cells expanded in bioreactor conditions after three serial passages. Statistics legend: *, **, ***, **** represent *P* ≤ 0.05, *P* ≤ 0.01, *P* ≤ 0.001, and *P* ≤ 0.0001, respectively

To further assess process robustness, additional commercially available hiPSC lines TC1133 and PLX1 were expanded through three serial passages in the PBS-0.1 Mini bioreactors using StemFlex and mTeSR1 media. Figure 6A and B show the growth kinetics of hiPSC lines TC1133 and PLX1 during the three serial passages, respectively. Each cell line maintained consistent cell growth patterns and rates through the three passages. As expected, there were some differences in inherent growth rates between cell lines; however, the trend in cell growth rate relative to media proved to be consistent with all three cell lines tested throughout this study. Specifically, StemFlex achieved higher cell densities throughout each culture when compared to mTeSR1. The average bioreactor day 6 cell densities when cultured in StemFlex medium for the 4YA, TC1133 and PLX1 cell lines were 0.75E6 ± (3.31E4), 1.53E6 ± (9.60E4), and 1.41E6 ± (3.52E4) viable cells/mL, respectively. When cultured with mTeSR1 medium, the average day 6 cell densities for the 4YA, TC1133, and PLX1 cell lines were 0.49E6 ± (5.14E4), 1.09E6 ± (8.50E4), and 0.85E6 ± (4.52E4) viable cells/mL, respectively. The consistency in cell growth is also shown in Fig. 6C and D where the overall specific growth rates at each passage for all cell lines (4YA, TC1133, and PLX1) in StemFlex and mTeSR1 are shown. Here it was evident that not only did the growth patterns remain consistent within the respective medium, the growth patterns between cell lines tested also remained consistent. That is, TC1133 had the fastest average growth rates in StemFlex and mTeSR1 at 0.030 h^− 1^ and 0.028 h^− 1^, respectively, and 4YA had the slowest average growth rates in StemFlex and mTeSR1 at 0.025 h^− 1^ and 0.022 h^− 1^, respectively. Importantly, for all cell lines grown in StemFlex and mTeSR1 media, the cumulative multiplication through the three bioreactor serial passages resulted in linear trendlines, as shown in Fig. 6E. This is an important characteristic of PSCs which, when cultured using the same PIVs and passaged using the same harvest protocol, should maintain consistent growth rates from one passage to the next. The use of StemFlex medium resulted in cumulative fold expansions of 4.27E5 and 3.52E5 for the TC1133 line and PLX1 line, respectively, after the serially passaged 3 consecutive cultures. The use of mTeSR1 medium resulted in cumulative fold expansions of 1.51E5 and 0.77E5 for the TC1133 line and PLX1 line, respectively. In addition to growth kinetics, normal aggregate morphology (i.e., spherical shape with smoothed and defined edges and lack of translucent cyst like structures) and a high degree of aggregate size homogeneity was observed throughout each bioreactor passage, with each additional cell line tested in both StemFlex and mTeSR1 media, as shown in Fig. 6F. At the end of the third bioreactor serial passage, cells were taken from both StemFlex and mTeSR1 bioreactor cultures to test the relative expression of pluripotent markers when compared to cells cultured in static controls. Relative expression of Oct4 was statistically higher in the cells from the bioreactor serial passage in StemFlex medium for both the TC1133 line and PLX1 line, as shown in Fig. 6G and H, respectively, compared to the control static culture cells. For cells cultured in mTeSR1, it was shown that there was no statistically significant difference of Oct4 expression with the TC1133 line (Fig. 6G), while there was statistically higher expression of Oct4 for the PLX1 line cultured through bioreactor serial passages compared to the static control (Fig. 6H). Relative expression of Sox2 was not statistically significant for either medium when observing the TC1133 line and PLX1 line, as shown in Fig. 6I and J, respectively. Taken together, these results demonstrate that pluripotency marker expression of Oct4 and Sox2 was either maintained or elevated after long-term bioreactor culture of iPSCs when compared to the static control.

## Discussion

Developing new media or enhancing existing media formulations in order to move from static planar vessels into dynamic suspension bioreactors or for improved bioprocess design are well documented in the literature, and knowledge regarding the most critical components in hPSC media has been greatly expanding throughout the last two decades [24]. This has led to a myriad of different media formulations for hPSCs [25–30]. Currently, there exists over 15 commercial media for hPSC culture [24]. However, most of these media formulations were developed with studies focused on optimizing media components and concentrations within static culture platforms and laboratory scale volumes [24, 26, 29]. The current study presents a novel comparison between five common commercial hPSC media (StemFlex, mTeSR1, CTS E8, PluriStem, and NutriStem) in a scalable suspension bioreactor, in which hPSCs grow as aggregates, through short-term and long-term culture. Although some of the commercial media formulations have been tested in various dynamic systems, specifically mTeSR1 [22, 31–36], most have no published results in suspension bioreactor platforms. Therefore, if a process has been developed using static culture vessels only at a small scale with a specified commercial medium, it is risky to assume that this medium will be translational to scalable suspension culture systems. Moreover, some studies tested PSC culture in suspension bioreactors only by inoculating cells from 2D planar seed train into the bioreactors and analyzing their growth without further passaging between bioreactors. Without data that demonstrates the performance of PSC media in suspension bioreactors, particularly through serial passages, efficiently translating protocols to clinical and commercial manufacturing systems and scales will likely be delayed.

This study demonstrated that, when using the VW bioreactor platform and bioprocess protocols outlined, hPSC expansion in those five commercial media tested herein could be successfully transitioned into a dynamic culture environment for a single bioreactor culture expansion period. The ability to transition all tested media successfully into dynamic culture using specified PIVs highlights the robust nature of the bioprocessing protocols used for this stage of the bioprocess workflow. This success was supported by normal aggregate formation, growth, and morphology along with relative consistencies in cell growth rates and maximum fold expansions achieved for all tested media. The latter is especially impressive as successful expansion has been defined in this study to be meeting or exceeding growth rates from top published reports in current literature [37–39]. Earlier studies in literature testing the translation of PSC expansion from static planar culture vessels into dynamic bioreactor systems report cell growth rates and fold expansions that are significantly lower [40–46] than recent publications [37–39]. It is important to note that in the current study there are some significant differences in the fold increase and average aggregate size between media tested through one bioreactor passage, especially by day 6 of culture. These differences are most likely due to the proprietary components and differences in their respective concentrations specific to the media. For example, NutriStem manufacturer notes there is a low level of basic fibroblast growth factor in their medium. This low concentration combined with the minimal feeding regime used in this 3D bioprocess protocol could contribute to the smaller aggregate diameters and lower fold-expansion results with NutriStem when compared to the StemFlex medium condition. It is, however, difficult to ascertain any conclusions without knowing the actual formulations of each different medium.

Although initial results from this study suggest that all five media tested could be transitioned from 2D to cultivate hiPSCs in 3D bioreactors, the results from the in-vessel dissociation of aggregates expanded in bioreactors and the serial bioreactor-to-bioreactor passage experiments highlighted the risks of drawing upon such conclusions prematurely or extending these conclusions to downstream protocols in the workflow. While some bioprocess workflows may require only one stage of PSC aggregate expansion in the bioreactor before starting differentiation, others will require multiple bioreactor-to-bioreactor passages to generate the required cell numbers for clinical or commercial manufacturing purposes, particularly for allogeneic therapies. For these bioprocess workflows it is critical to evaluate bioprocess harvesting/passaging protocols and expansion in 3D culture for prolonged durations. Most published literature that includes studies with PSC serial passaging in 3D culture systems were designed at a small-scale, and methods used to dissociate aggregate samples from the bioreactor for subsequent passages are not scalable [47, 48]. As such, we tested our previously optimized in-vessel aggregate dissociation protocol to harvest the entire bioreactor volume for serial passaging [5].

When this in-vessel dissociation protocol was employed on the aggregates that had been expanded for 6 days in the various media tested, significant differences were noted between the different media. When dissociating aggregates cultured in StemFlex or mTeSR1, harvest recoveries were ~ 100% with no noticeable cellular debris. In comparison, when dissociating aggregates cultured in CTS E8, PluriStem, or NutriStem, there were significantly lower harvesting efficiencies and a decrease in the cell quality. Most notably, the dissociation process resulted in cell lysis as indicated by the visible DNA strands and cellular debris in the supernatant. While the viabilities of the cells post in-vessel dissociation of the aggregates expanded in bioreactors using CTS E8, PluriStem and NutriStem were lower than observed during the pre-harvest cell sample count, these differences alone do not account for the significant reduction in cell number obtained from the harvest. It is presumed that these lysed strands account for a large fraction of cells that could not be counted post in-vessel dissociation. Since the lysed DNA is known to be a sticky cell substance, it is likely that these strands caused some of the aggregates and single cells to adhere together during the dissociation procedure. These agglomerated cell-aggregate masses were excluded from the recovery of the dissociated single cells from the bioreactor harvest, and these cell losses would account for the large decrease in total cell number post in-vessel dissociation. These results indicate that the choice of media not only impacts cell culture process and quality output variables such as growth rate, aggregate morphology, and aggregate size, but it can have a significant impact on cell harvesting/passaging process parameters and results. Such process steps, including in-vessel aggregate dissociation, are often not considered when designing and testing upstream cell culture processes. The current study highlights the importance of evaluating all steps of the bioprocess (expansion, dissociation/harvesting, and passaging) to truly encapsulate the impact of different input variables.

The different outcomes of aggregate dissociation into viable single cells were most likely due to media components and their impact on cell quality during the first bioreactor expansion phase. For example, shear protectants, such as Pluronic, are commonly added to cell culture media to prevent cell damage or death caused by fluid shear and aeration in agitated suspension bioreactors [49, 50]. As such, the absence of these components can profoundly impact cell quality when cells are exposed to the hydrodynamic environments in bioreactors [44, 51–53]. In our previous studies we have shown that there is an optimal range of hydrodynamic values, and thus agitation rates, for the expansion of high quality hiPSC aggregates in the VW bioreactors. However, to facilitate the dissociation of aggregates into single cells during the in-vessel dissociation process, higher shear forces are required than what we recommended for the culture expansion phase [20]. For the optimized in-vessel aggregate dissociation protocol, a reduced working volume of 20 mL and an agitation rate of 80 rpm was effectively used in the PBS-0.1 Mini bioreactors. This falls outside the suitable hydrodynamic range for hiPSC expansion culture. Under this in-vessel dissociation protocol, the cells are subjected to turbulent fluid conditions where eddies generated by hydrodynamic forces are smaller than aggregate diameters, thus causing aggregates to be sheared apart into single cells. Although these higher forces are required to effectively dissociate the aggregates, it may leave the cells more susceptible to damage. As such, having the cells exposed to shear protectants throughout the culture period, priming them for high shear dissociation, may be necessary to preserve cell quality and viability. Therefore, it may be possible to increase the success of a complete bioprocess in the VW bioreactors when using CTS E8, PluriStem, and NutriStem if they are supplemented with such protectants. If these media are considered for the culture of PSCs in suspension bioreactors, this hypothesis should be verified by testing such shear protectants at various concentrations.

In addition to the immediate observations noted from the in-vessel aggregate dissociation studies, the long-term impacts of the media tested on growth and quality outputs were observed during the subsequent serial passages. It should be noted that there appears to be an extended lag phase present for all media conditions during the second bioreactor passage. Comparing day 1 and day 2 average cell counts indicates they are lower during the second bioreactor culture compared to the first bioreactor culture for all media conditions. This apparent lag could be a result of cell exhaustion at the end of the first bioreactor culture. If the cells were nearing the limits of a factor like dissolved oxygen concentration, pH buffer capacity or nutrient availability, a portion may start to exit the exponential growth phase undergoing proliferation at a reduced rate and then enter a stationary phase. These cells that exit the exponential growth phase are likely to display an increased lag phase in the following passage before re-entering the exponential growth phase. Another observation that was made was the cells cultured in either StemFlex or mTeSR1 maintained consistent growth through three bioreactor passages. Conversely, cells cultured in CTS E8, PluriStem, or NutriStem showed a significantly decreased growth and changes to aggregate morphology, specifically during the third bioreactor expansion culture where there was negligible growth over 6 days. This aligns with the findings from the aggregate dissociation stage of the first bioreactor expansion culture where the aggregates expanded in StemFlex and mTeSR1 were almost completely dissociated into viable single cells with ~ 100% harvest efficiency whereas the cultures with CTS E8, PluriStem, and NutriStem resulted in macroscopically visible cellular debris and reduced viable cell recovery during the passage. The challenges revealed during the in-vessel dissociation-mediated harvest process impacted results in the subsequent passages. Although cells cultured in CTS E8, PluriStem, and NutriStem were able to recover and show some growth in the second passage, by the third passage the cultures experienced extended lag phases with little to no cell recovery in growth. This was mostly likely an indication of compounded cell quality reduction through the serially passaged culture in bioreactors. These amplified consequences again highlight the importance of extended bioprocess testing timelines to identify protocols which will produce clinically or commercially relevant numbers of high quality and functional PSCs.

While key process attributes including growth kinetics, aggregate morphology, and viability are critical in assessing bioprocess robustness, assays that test genetic stability and pluripotency are essential to assessing the maintenance of PSC cell quality and function. It has been noted in literature that the use of enzymatic passaging can increase the risk of genetic instability [28, 54, 55]. Therefore, to validate the success of the overall bioprocess, cell quality should be further analyzed. Of note, the mTeSR1-based culture tested with the 4YA cell line and bioprocess PIVs from our past publications was taken as a positive control when establishing genetic stability and pluripotency maintenance in this study. In our previous publications we have performed in depth genetic (G-banding karyotyping), phenotypic (surface and nuclear marker cell-image staining, polymerase chain reaction, and flow cytometry) and functional (tri-lineage directed differentiation and teratoma formation) testing following serial passages in the VW bioreactor [5, 21]. These studies demonstrated the robust nature of the bioprocess to generate high quality PSCs, allowing the focus of this study to be on evaluating other critical input and output variable relationships throughout a long-term bioprocess.

Throughout the short- and long-term media comparison studies presented here, it was found that, in addition to the mTeSR1 process control condition, the condition cultured with StemFlex medium was successful in maintaining key process attributes (i.e., cell viability/growth kinetics and aggregate size/morphology) throughout the bioprocess. As such, we continued to passage the hiPSCs cultured in StemFlex medium through a total of 10 bioreactor serial passages (60 days) to demonstrate extended bioprocess robustness. During this prolonged testing, not only did the cells maintain growth characteristics and aggregate morphology throughout each passage, but they were also found to maintain genetic stability and pluripotent function. Cell samples from the StemFlex bioprocess condition were taken at the end of the third and tenth bioreactor serial passage for G-banding and teratoma formation assays. At both time points, genetic stability and pluripotent function in-vivo were confirmed. This again highlighted the robust nature of the developed protocol to produce quality-assured cells. It is recognized in literature that more subtle changes in genetic stability and pluripotency may not be distinguishable using these traditional quality assays [55]. As such, higher resolution quality assays should be employed before finalizing the bioprocess used in clinical or commercial manufacturing settings to ensure safety and efficacy.

iPSC line-to-line variability is another prominent challenge in bioprocess development [44]. As such, in the final part of this study we repeated the long-term serial passage testing with the successful media candidates (StemFlex and mTeSR1) using two additional hiPSC lines from different commercial vendors. The consistent patterns and predictable results of cell growth and aggregate morphology demonstrated in these final experiments further highlight the robust nature of the bioprocess protocols established. Differences in the growth rates throughout the three serial passages using each cell line were shown to be minimal. All conditions demonstrated exponential cumulative multiplication patterns through the three serial passages and normal aggregate growth and morphology. In addition, reverse transcription qPCR results for pluripotency markers Oct4 and Sox2 demonstrated that the cells had either maintained or elevated expression levels after long-term bioreactor culture when compared to static control. These results build further confidence in the bioprocessing protocols established and provide evidence that these protocols can be translated to other hiPSC lines with minimal optimization required.

## Conclusions

In the development of bioprocesses for the expansion of pluripotent cells, it is critical to systematically evaluate different PIVs as they can profoundly impact output parameters either independently or synergistically with other variables. In this study, a previous optimized bioprocessing protocol was assessed for process robustness for the expansion of hiPSCs in a dynamic culturing environment. Further, this protocol was used to assess the impact of cell culture media – one of the most critical PIVs in the development of a bioprocess. As such, five commercially available media were evaluated for both short- and long-term expansion of hiPSCs using the PBS-0.1 Mini VW bioreactor system. It was demonstrated that all tested media could be successfully transitioned to dynamic culture indicating the robust nature of the bioprocessing protocols in the VW bioreactor platform. However, long-term culture experiments highlighted the importance of such studies to truly assess bioprocess robustness. Specifically, when subjected to in-vessel aggregate dissociation procedures and subsequent passages, some of the tested PSC media resulted in significant decreases in cell growth and quality that was not observed in the initial screening. Conversely, these studies showed that StemFlex and mTeSR1 media could be effectively used for long-term culture of hiPSCs in scalable suspension bioreactors, which was validated using three commercial hiPSC lines. Through this study, we have demonstrated the importance of rigorous design and evaluation in the development of robust bioprocesses to achieve clinically and commercially relevant PSC populations.

## Data Availability

The datasets generated and/or analysed during the current study are available from the corresponding author on reasonable request.

## Abbreviations

BSC: Biosafety cabinet
hiPSC: Human induced pluripotent stem cell
hPSC: Human pluripotent stem cell
IHC: Immunohistochemistry
iPSC: Induced pluripotent stem cell
MX: Medium exchange
P1: Passage 1
P2: Passage 2
P3: Passage 3
PIV: Process input variable
POV: Process output variable
PSC: Pluripotent stem cell
RPM: Revolutions per minute
RT-qPCR: Reverse Transcription Quantitative Polymerase Chain Reaction
VW: Vertical-Wheel^®^
